# An Orthotopic Resection Surgical Technique Using an Inferior Infracolic Approach for Laparoscopic Pancreaticoduodenectomy

**DOI:** 10.3390/jcm12020590

**Published:** 2023-01-11

**Authors:** Yutong Yao, Junjie Xiong, Ziyao Wang, Xing Wang, Xubao Liu, Nengwen Ke

**Affiliations:** 1 Department of Pancreatic Surgery, West China Hospital, Sichuan University, No. 37, Guo Xue Alley, Chengdu 610041, China; 2 Liver Transplantation Center and HBP Surgery, Sichuan Cancer Hospital & Institute, Sichuan Cancer Center, Cancer Hospital Affiliate to University of Electronic Science and Technology of China, Chengdu 610042, China

**Keywords:** laparoscopic pancreaticoduodenectomy, no-touch isolation, inferior infracolic approach

## Abstract

The no-touch isolation technique has been widely used in cancer surgery as a strategy to prevent cancer cells from spreading; however, it is difficult to apply in laparoscopic pancreaticoduodenectomy (LPD). Here, we describe an orthotopic resection surgical technique that applies a no-touch principle for LPD and can help with the in situ resection of tumors. In implementing this surgical strategy, Kocher’s maneuver was not performed first. Instead, after the exploration of the abdominal cavity, the distal stomach and the pancreatic neck were transected. Then, the dissection of the uncinate process of the pancreas, the duodenum, and the superior mesenteric vein and artery is carried out via an inferior infracolic approach. Finally, the pancreatic head and duodenum were removed in situ. Among the 41 patients who underwent this technique, two (4.9%) required conversion to open surgery due to uncontrolled bleeding. The average operative time was 335 min (248–1055 min). The mean estimated blood loss was 300 mL (50–1250 mL). Two patients (4.9%) underwent combined PV resection and reconstruction; six patients (14.6%) required a blood transfusion; two patients (4.9%) suffered from postoperative bleeding; two patients (4.9%) suffered from Grade B pancreatic fistulas; one patient (2.4%) suffered from bile leakage; and three patients (7.3%) suffered from abdominal fluid collection. No patients died during the perioperative period. Therefore, orthotopic LPD using an inferior infracolic approach is safe and feasible for patients with malignant pancreatic head and periampullary tumors. However, further investigations are required to elucidate its oncological benefits.

## 1. Introduction

Pancreatoduodenectomy (PD) is still one of the most effective methods for removal of malignant pancreatic head and periampullary tumors. In conventional PD, Kocher’s maneuver, which is used to mobilize the pancreatic head and duodenum, must be carried out prior to dissection of the surrounding vessels. In this process, the specimen serves as “a convenient handle for retraction” by the surgeon; however, squeezing the tumor may increase the risk of shedding cancer cells into the PV, lymphatic vessels, and peritoneal cavity [1,2]. The no-touch isolation technique has been widely used in cancer surgery as a strategy to prevent cancer cells from spreading [3,4,5]. Some researchers have attempted to use this technique in open PD, showing that it can significantly reduce the circulating tumor cell rate [1,6,7], but it is difficult to apply in laparoscopic pancreaticoduodenectomy (LPD) because of technical difficulties. In conventional LPD, the anterior surface of the pancreatic head and the horizontal segment of the duodenum in the superior colic region are usually dissected and displayed. If Kocher’s maneuver is not carried out, the uncinate process of the pancreas and the ascending segment of the duodenum cannot be fully exposed due to obstruction from the transverse colon, transverse mesocolon, and middle and right blood vessels of the colon. It is then difficult to dissect the blood vessels that run in and out of the uncinate process of the pancreas, including the inferior pancreaticoduodenal artery (IPDA) and inferior pancreaticoduodenal vein (IPDV), which can lead to uncontrollable bleeding. Inspired by the intermediate approach for laparoscopic right hemicolectomy, we developed an orthotopic resection surgical technique using an inferior infracolic approach for LPD. Here, we describe the surgical procedure with images and present the perioperative results regarding the use of the technique.

## 2. Patients and Methods

### Patients

We began to perform LPDs using this technique in October 2019. Forty-one cases of LPDs were included in this study from October 2019 to September 2021. The inclusion criteria were (1) pancreatic head cancer or periampullary cancer; (2) preoperative CT or MRI evaluation showing that the tumor was resectable (for example, there was no IVC or SMA invasion and no lymph node metastasis in 16 groups); and (3) no variation in the origin of the right hepatic artery in preoperative CT or MRI evaluation, such as SMA. Data on the demographic characteristics (age, sex, BMI, American Society of Anesthesiology, preoperative biliary drainage, and pathologic diagnosis), operative outcomes (conversion to open surgery, operative time, estimated blood loss, combination with portal vein resection and reconstruction, and transfusion), and postoperative results (postoperative hospital stay, time to first passage of flatus, time to first out-of-bed activity, perioperative mortality, and complications) from the cases were prospectively collected and retrospectively analyzed. All patients were informed about the possible advantages and disadvantages of this study, and signed consent forms were obtained. This study was conducted according to the Declaration of Helsinki principles, and ethical approval was obtained from The Clinical Medical Research Ethics Committee of Sichuan University (2019735).

## 3. Surgical Procedures

### 3.1. Patient Position and Trocar Distribution

**Figure 1:**
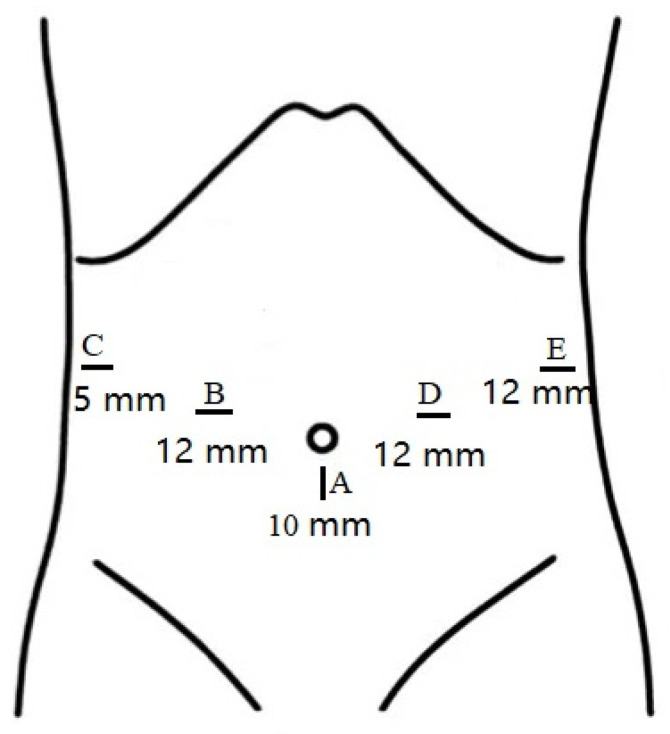
Sizes and distributions of trocars.

The patients were placed in the supine position with their legs separated. Five trocars were symmetrically distributed in all patients. One 10 mm trocar was used for the laparoscope port, which was located at the inferior umbilicus; two 12 mm trocars were located at the right and left midclavicular lines; and one 12 mm trocar and one 5 mm trocar were distributed at the left and right anterior axillary lines, respectively (Figure 1).

### 3.2. Orthotopic Resection Surgical Technique

#### 3.2.1. Exploration and Initial Processing

**Figure 2:**
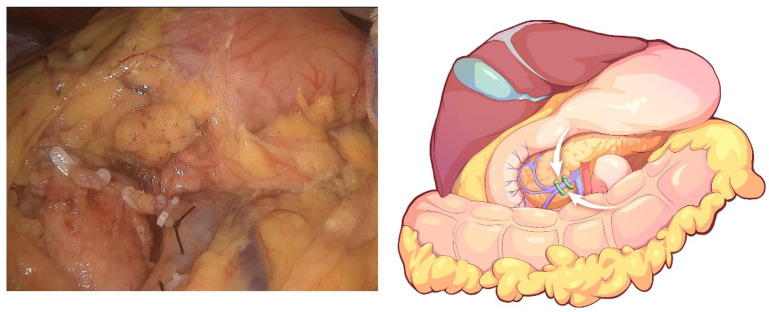
Dissection of Henle’s trunk.

**Figure 3:**
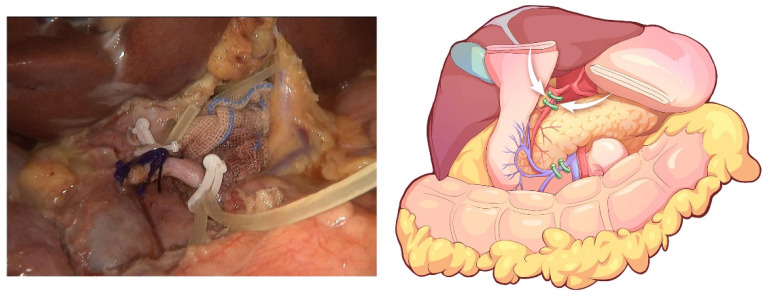
The GDA is identified along the common hepatic artery (CHA) and clipped with Hem-o-lock after thread ligation.

The initial step of the operation is the exploration of the whole abdominal cavity, especially the liver and peritoneum, to preliminarily assess the tumor stage and resectability. For this, a “liver retraction line” is used to expose the pancreatic and hepatic portal by a blue purse string with two straight needles at both ends. Specifically, a straight needle was used to puncture into the abdominal cavity through one side of the abdominal wall (generally the left upper abdomen) and then puncture into the body through the other side of the abdominal wall (generally the right upper abdomen). A segment of line in the abdominal cavity was clipped with the lesser omentum with Hem-o-lock. Finally, the left liver was retracted after the two ends of the line are tightened outside the abdominal cavity. This method is often used in pancreatic laparoscopic surgery to expose the pancreatic and hepatic portal. In conducting this operation, we also aimed to facilitate the suspension of the transverse colon in the next step. Afterward, the greater omentum was dissected between the greater curvature of the stomach and the omentum, and the neck and body of the pancreas were displayed. The superior mesenteric vein (SMV) was identified and exposed at the ventral margin of the pancreatic neck. The right gastroepiploic vein and Henle’s trunk were dissected (Figure 2). The distal stomach was transected 3–5 cm away from the pylorus using a linear stapler, and the No. 8a lymph node was dissected. Then, the gastroduodenal artery (GDA) was identified along the common hepatic artery (CHA) and clipped with Hem-o-lock after thread ligation (Figure 3). The pancreatic neck was transected over the SMV with an ultrasonic scalpel, and the main pancreatic duct was transected using cold scissors.

#### 3.2.2. Procedures of the Inferior Infracolic Approach

**Figure 4:**
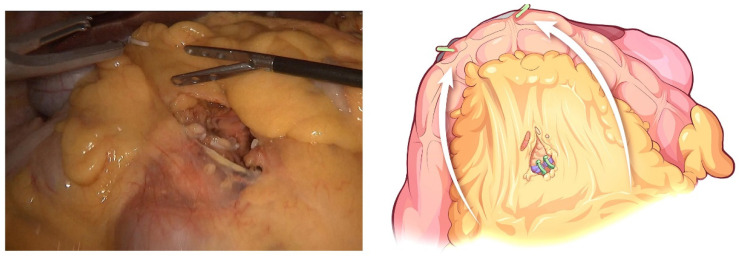
The transverse colon is suspended by clipping its mesocolon to the gallbladder and the “liver retraction line”.

**Figure 5:**
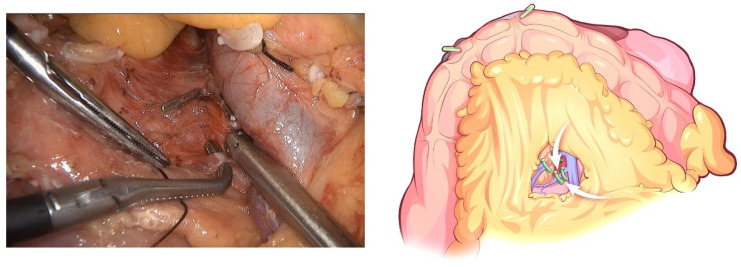
The IPDV being gently ligated in situ.

**Figure 6:**
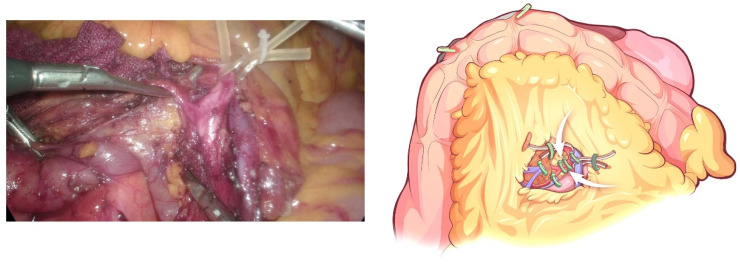
Identification and ligation of the IPDA.

**Figure 7:**
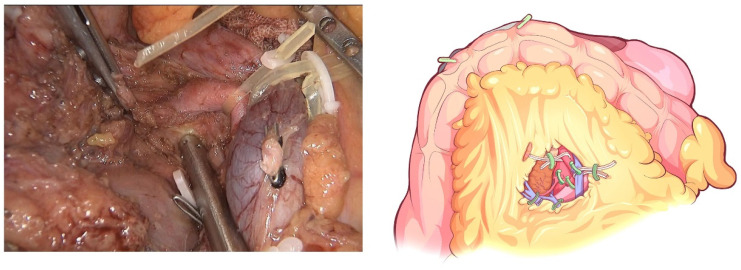
Complete clearance on all tissues located 180 degrees to the right of the SMA is performed to achieve a negative margin.

**Figure 8:**
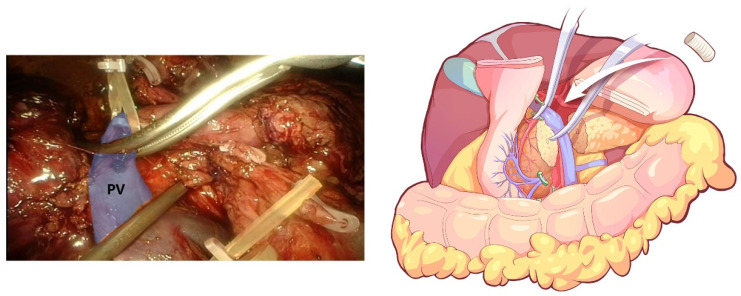
End-to-end anastomosis, which was performed after combined resection of the involved PV.

The transverse colon was suspended by clipping the mesocolon to the gallbladder and the “liver retraction line” (Figure 4). The transverse mesocolon was opened, and the inferior part of the duodenum was mobilized to display the main SMV and the first branch of the jejunal vein. The IPDV was gently ligated in situ (Figure 5). The main SMV was secured with a vessel loop. The superior mesenteric artery (SMA) was identified and displayed when the SMV was retracted to the left. The SMA was secured with a vessel loop. The IPDA was ligated after being clipped with Hem-o-lock (Figure 6). Then, the vascular sheath of the SMA was dissected to the root in the cranial direction. In this process, we performed complete clearance on all tissues located 180 degrees to the right of the SMA to achieve a negative margin (Figure 7). The superior pancreaticoduodenal vein (SPDV) was ligated. The jejunum was transected 15 cm away from the ligament of Treitz using a linear stapler. The mesentery of the proximal jejunum and the ligament of Treitz were incised, and the proximal jejunum was retracted to the right through the tunnel behind the SMA. The inferior vena cava (IVC) and left renal vein (LRV) were displayed when the mesentery of the uncinate process was dissected. If the tumor had approximated or invaded the PV, we performed PV/SMV resection or reconstruction by total laparoscopy (Figure 8). For this, after the uncinate process was completely dissected, the SMV and PV were occluded using laparoscopic bulldog clips. The extent of PV/SMV involvement was then assessed. For cases with vascular lateral wall involvement (<90°), wedge resections were performed using cold scissors, and the defect was repaired with a running suture. For cases with extensive vascular involvement, a circular resection of the involved venous segment was performed. Moreover, for cases with vascular involvement less than 3 cm, end-to-end anastomoses were performed. Grafts were required for cases with vascular involvement greater than 3 cm. In general, 5-0 Prolene was used for vascular anastomosis, and a running suture was preferred.

#### 3.2.3. Reversed Kocher’s Maneuver and Lymphadenectomy

**Figure 9:**
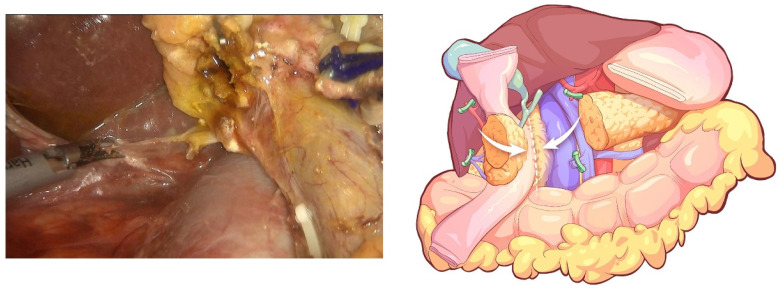
A reversed Kocher’s maneuver alongside the IVC to complete resection of the posterior peritoneum of the duodenum and pancreatic head.

The transverse colon was restored, the gallbladder was dissected, and the common bile duct was transected. A reversed Kocher’s maneuver alongside the IVC completed the resection of the posterior peritoneum of the duodenum and pancreatic head (Figure 9). A standard lymphadenectomy (No. 12a, 12p, 8, 9) was performed along the PV and CHA to the celiac trunk. The specimen was placed in a disposable bag and removed from the enlarged umbilical incision after reconstruction of the digestive tract.

#### 3.2.4. Reconstruction of Digestive Tract

A classical child anastomosis was adopted, and a pancreaticojejunostomy was performed using end-to-side and duct-to-mucosa maneuvers and an intermittent suture with 4-0 absorbable line; a support tube was retained in pancreatic duct. The pancreatic parenchyma and the seromuscular layer of the jejunum were intermittently sutured with 4-0 absorbable thread. An end-to-side anastomosis was performed with 4-0 absorbable line for biliary and intestinal reconstruction. The gastrojejunostomy was reconstructed with a linear cutting occluder, and the common gastrointestinal opening was closed with 3-0 sutures.

## 4. Results

The demographic characteristics of our case series are shown in Table 1. This study included 41 cases of LPDs (20 male patients and 21 female patients) that underwent the orthotopic resection surgical technique using an inferior infracolic approach. The median age of these patients was 56 years (23–83 years). The median body mass index (BMI) of the patients was 23.4 kg/m^2^ (16.6–28.6 kg/m^2^). The postoperative pathological diagnoses included ampullary adenocarcinoma (6 cases), pancreatic ductal adenocarcinoma (25 cases), pancreatic neuroendocrine tumor (4 cases), distal cholangiocarcinoma (3 cases), solid pseudopapillary tumor (2 cases), and other (1 case).

The surgical outcomes and postoperative details of our case series are shown in Table 2. Two patients (4.9%) in our series required conversion to open surgery due to uncontrolled bleeding. One patient (2.4%) in this group underwent reoperation. The median operative time was 335 min (248–1055 min), and the median estimated blood loss was 300 mL (50–1250 mL). Two patients (4.9%) underwent PV resection and reconstruction combined. Six patients (14.6%) required blood transfusion. The median postoperative hospital stay was 12 days (3–125 d). The median time to the first passage of flatus was 3 days (1–5 d), and the median time to the first out-of-bed activity was also 3 days (2–7 d). Two patients (4.9%) suffered from postoperative bleeding. According to the ISGPS definition [8], one patient (Grade B) suffered from abdominal bleeding due to a gastroduodenal artery pseudoaneurysm on the 21st postoperative day. This patient underwent angiography and arterial embolization therapy. Another patient (Grade C) underwent an angiography on the fourth day after operation due to abdominal bleeding, and no obvious abnormality was found; the bleeding stopped after the examination. On the fifth day after the interventional procedures, the patient underwent a laparotomy due to rebleeding. A pseudoaneurysm of the left hepatic artery was found to be ruptured, and the left hepatic artery was ligated. No obvious abnormality was found in liver function during monitoring after the operation. According to the new ISGPS definition [9], 13 patients (31.7) suffered from biochemical leakage, and two patients (4.9%) suffered from Grade B pancreatic fistulas. One patient (2.4%) suffered from bile leakage; following the full drainage of the abdominal drainage tube (<50 mL/d), the patient did not exhibit abdominal infection or fluid accumulation. The abdominal drainage tube was removed on the tenth day after the operation. Three patients (7.3%) suffered from abdominal fluid collection, of which two required percutaneous drainage. No patients died during the perioperative period.

## 5. Discussion

Here, we present a technique for laparoscopic in situ pancreatoduodenectomy resection. The technique is considered safe based on the perioperative results. Compared with conventional pancreatoduodenectomy, there is no increase in the risk and duration of surgery. In addition, the technique implements the no-touch principle.

The no-touch isolation technique was originally used to prevent seeding and cancer cell dissemination in colon cancer surgery and can reduce the risk of tumor metastasis via PV [10,11]. The technique is also reported to be effective in open PD surgery [1,3,7,12]. Several studies have found that cancer cells can be detected in droplets from resected tissues and the pancreatic bed in PD [1,13]. Gall et al. reported that circulating tumor cells (CTCs) could be detected in the PV circulation of patients with pancreatic ductal adenocarcinoma (PDAC) and that their count increased following tumor manipulation during conventional PD. Compared with the conventional operation group, the CEA mRNA in the portal vein blood of the no-touch isolation technique group was significantly reduced after tumor manipulation, and the recurrence rate also showed the same trend; meanwhile, the mean survival time and 3-year survival rate increased significantly [6,13,14]. Based on the above findings, we conclude that it is safer and more effective to perform pancreaticoduodenectomies using the no-touch technique in patients with malignant pancreatic head and periampullary tumors. We also noticed that there are several researches report using of the “no-touch isolation” principle in LPD surgery [15,16,17]. The authors of these also fully realized the importance of the “no-touch isolation” principle in pancreaticoduodenectomy for patients with pancreatic cancer. However, various approaches were used, such as the subcolonic mesocentric approach, the left posterior SMA approach, and the supercolic medium superior SMA approach, which resulted in a lack of standardization and unity. We believe that in the process of resection, as long as adequate transverse colon suspension is carried out, resection can be completed using an inferior infracolic approach.

We consider this technique to have the following advantages: (i) The pancreatic head is not manipulated prior to dissection of the vessels around the pancreatic head. This kind of operation, from shallower to deeper depths, is in accordance with the basic principle of oncology surgery. (ii) From the laparoscopic perspective, there is no need to cross the transverse colon after suspension. There is therefore no occlusion between tissues, organs, and blood vessels regarding the surgeon’s vision, allowing full exploitation of the advantages of laparoscopy, which is conducive to thorough dissection of the peripheral nerves and lymph nodes. (iii) When displaying the ascending segment of the duodenum, there is no need to dissect the descending colon, which saves operation time. (iv) The ascending segment of the duodenum and the first branch of the jejunum vein can be used as anatomical landmarks to identify the SMA and IPDA, which can reduce the risk of operative bleeding. The two key points of our technique are as follows: (i) During the exploration and initial processing, the SMV branch and arterial blood supply are first ligated, and the PV branch is then dissected after suspension of the transverse colon. Thus, using this procedure, bleeding can be reduced by avoiding congestion in the pancreaticoduodenal region. In addition, repeatedly reversing the colon can be avoided, thus saving operation time. (ii) The gallbladder is not removed first, allowing convenient suspension of the transverse colon by clipping it to the gallbladder.

The longest operation duration in our series was 1055 min. The patient was a 63-year-old female with a BMI of 28.3 kg/m^2^. She underwent a laparoscopic biliary exploration due to “common bile duct stones”. During the operation, severe abdominal tissue adhesion was found, and chronic pancreatitis was also observed in the pancreas. During the operation, after closing the trocar hole and when the patient had not recovered from anesthesia, it was found that about 50 mL of blood had flowed out of the abdominal drainage tube. Laparoscopic exploration was immediately performed in the operating room, and a hemorrhage was found. After laparoscopic exposure and hemostasis, the bleeding did not stop. After 2 h, the decision was made to switch to open surgery. Following abdominal cavity resection, an open pancreaticoduodenal anastomosis was conducted, during which hemorrhaging was observed in the branches of splenic veins behind the pancreas. After hemostasis by suture and ligation, pancreaticoduodenal anastomosis was performed again. After the operation, we discussed that it was a very difficult pancreatoduodenectomy; however, if we had initially decided to proceed directly open surgery, it may have also been equally time-consuming. Our experience and the lesson learned from the operation here is that if, during the operation, the bleeding site is deep and the hemostasis effect is not ideal, the anastomosis should be removed as soon as possible, fully exposed, and explored to reduce both the hemostasis time and surgical risk. In the first ten cases of surgery, we found that the anatomy of the uncinate process of the pancreas and the branches of the SMV were still sites with a high risk of bleeding, especially in cases of obesity, chronic pancreatitis, and previous abdominal surgery. As a result of this new principle, the operation process and vascular anatomy position differ compared with in the traditional operation, which increases the difficulty of the operation to a certain extent and requires good cooperation, skills, and anatomical knowledge from the operation team. We found that patients with pancreatic head cancer or periampullary cancer often presented with obstructive jaundice, hypoproteinemia, malnutrition, and other conditions before surgery, and they were unable to orally consume for a period of time after surgery. Therefore, we routinely placed a nasojejunal nutrition tube through the nose during the operation and positioned the distal end of the nutrition paste at about 15 cm from the distal end of the gastrointestinal anastomosis such that the enteral nutrition liquid could be continuously pumped from the first day after the operation. In addition, we also routinely injected somatostatin into the vein 3 days after surgery to reduce the incidence of anastomotic leakage. Therefore, the patient could only move in bed 3 days after surgery, including rolling, half sitting, breathing exercises, lower limb muscle exercises, etc.

We acknowledge that this surgical technique is not suitable for all pancreatic head and periampullary cancers. The most suitable indication is resectable cancer on preoperative evaluation. Presently, the sensitivity and specificity of a high-magnetic field MRI system and dynamic contrast-enhanced multislice CT in the diagnosis of resectability of pancreatic cancer can reach 100% and 93.3%, respectively, which makes it possible to accurately identify the resectability of pancreatic cancer before surgery. For borderline resectable pancreatic head cancer, an “artery first” approach still has significant advantages in judging resectability and improving the R0 resection rate; these patients may also need systemic treatment, such as neoadjuvant chemotherapy. In addition, it is still necessary to perform Kocher’s maneuver in cases of suspected IVC invasion or No. 16 lymph node metastasis because early transection of the stomach and pancreatic neck may lead to R1 resection.

It should be emphasized that with this new technique, the operation process and the anatomic position of blood vessels differ from those of the traditional operation, which increases the difficulty of the operation to a certain extent; thus, good cooperation, skills, and anatomical knowledge are required from the operation team. We suggest that this method can be performed by surgeons who have passed the LPD learning curve.

## 6. Conclusions

According to our experience, we suggest an orthotopic resection surgical technique utilizing an inferior infracolic approach for LPD that can help resect tumors in situ and en bloc. We demonstrate that orthotopic LPD via an inferior infracolic approach is safe and feasible for patients with malignant pancreatic head and periampullary tumors. However, further investigation is required to determine its oncological benefits.

## Figures and Tables

**Table 1:** Demographic characteristics.

Variables	
Cases	41
Age (median (interquartile range)) (y)	56 (23–83)
Sex (male/female)	20/21
BMI (median (interquartile range)) (kg/m^2^)	23.4 (16.6–28.6)
American Society of Anesthesiology	
II	36
III	5
Preoperative biliary drainage	2
Pathologic diagnosis	
Ampullary adenocarcinoma	6
Pancreatic ductal adenocarcinoma	25
Pancreatic neuroendocrine tumor	4
Distal cholangiocarcinoma	3
Solid pseudopapillary tumor	2
Other	1

**Table 2:** Surgical outcomes and postoperative details.

Variables	
Conversion to open surgery (n, %)	2(4.9)
Reoperation (n, %)	1 (2.4)
Operative time (min) *	335 (300–1055)
Estimated blood loss (mL) *	300 (50–1250)
Combined with portal vein resection and reconstruction (n, %)	2 (4.9)
Transfusion (n, %)	6 (14.6)
Postoperative hospital stay (d) *	12 (3–125)
Time to first passage of flatus (d) *	3 (1–5)
Time to first out-of-bed (d) *	3 (2–7)
Perioperative mortality (%)	0 (0)
Complications (n, %)	
Clavien–Dindo classification (n, %)	
Grade I	0(0)
Grade II	3(7.3)
Grade IIIa	3(7.3)
Grade IIIb	1(2.4)
Grade IVa	0(0)
Grade IVb	0(0)
Grade V	0(0)
Postoperative bleeding	
Grade A	0(0)
Grade B	1(2.4)
Grade C	1(2.4)
Pancreatic fistula	
Biochemical leakage	13(31.7)
Grade B	2 (4.9)
Grade C	0 (0)
Bile leakage	1 (2.4)
Abdominal fluid collection	3 (7.3)

* Data are expressed as the median and interquartile range.

## Data Availability

All data generated or analyzed during this study are available from the corresponding author on reasonable request.
